# Difference between preferred and non-preferred leg in peak speed, acceleration, and deceleration variables and their relationships with the change-of-direction deficit

**DOI:** 10.1038/s41598-022-26118-w

**Published:** 2022-12-12

**Authors:** Ana Filipa Silva, Rafael Oliveira, Javier Raya-González, Daniel van den Hoek, Zeki Akyildiz, Mehmet Yıldız, Joel M. Garrett, Hadi Nobari, Filipe Manuel Clemente

**Affiliations:** 1 Escola Superior Desporto e Lazer, Instituto Politécnico de Viana do Castelo, Rua Escola Industrial e Comercial de Nun’Álvares, 4900-347 Viana Do Castelo, Portugal; 2 Research Center in Sports Performance, Recreation, Innovation and Technology (SPRINT), 4960-320 Melgaço, Portugal; 3 The Research Centre in Sports Sciences, Health Sciences and Human Development (CIDESD), 5001-801 Vila Real, Portugal; 4 Sports Science School of Rio Maior–Polytechnic Institute of Santarém, 2040-413 Rio Maior, Portugal; 5 Life Quality Research Centre, 2040-413 Rio Maior, Portugal; 6 Faculty of Health Sciences, Universidad Isabel I, Burgos, Spain; 7 School of Behavioural and Health Sciences, Australian Catholic University, Brisbane, QLD Australia; 8 Sports Science Department, Gazi University, Ankara, Turkey; 9 Afyon Kocatepe University Sports Science Faculty, Afyonkarahisar, Turkey; 10 Department of Exercise Physiology, Faculty of Educational Sciences and Psychology, University of Mohaghegh Ardabili, Ardabil, 5619911367 Iran; 11 Faculty of Sport Sciences, University of Extremadura, 10003 Cáceres, Spain; 12 Department of Motor Performance, Faculty of Physical Education and Mountain Sports, Transilvania University of Braşov, 500068 Brasov, Romania; 13 Delegação da Covilhã, Instituto de Telecomunicações, 1049-001 Lisbon, Portugal; 14 School of Health and Behavioural Sciences, University of the Sunshine Coast, Queensland Sippy Downs, Australia; 15 School of Health Sciences and Social Work, Griffith University, Queensland Gold Coast, Australia

**Keywords:** Physiology, Medical research, Engineering

## Abstract

The aim of this study was two-fold: (i) analyze the variation of kinematic measures between using preferred and non-preferred legs while braking during the 5–0–5 change of direction test; and (ii) test the relationships between kinematic measures, and change-of-direction deficit (CODD). A cross-sectional study using twenty adult male soccer players (21.6 ± 2.0 years; 73.2 ± 6.1 kg; 174.8 ± 4.5 cm) was employed. Players performed three repetitions of the 5–0–5 test using each leg during the braking phase. Players have used the Polar Team Pro to obtain the kinematic measures of peak speed, peak acceleration, and peak deceleration. Additionally, the CODD was also obtained using single-beamed photocells. Comparisons revealed a significantly greater peak acceleration (+ 0.22 m/s^2^; *p* < 0.001) and deceleration (+ 0.17 m/s^2^; *p* = 0.004) for the non-preferred leg. There were no significant correlations were found between CODD and peak accelerations (r = − 0.014, [− 0.193; 0.166]), peak decelerations (r = − 0.052, [− 0.229; 0.128]) or peak speed (r = 0.118, [− 0.063; 0.291]). This study revealed that preferred and non-preferred leg must be analyzed differently since they are different in the kinematic variables. However, CODD seems independent of leg preference and the kinematic measures of a 5–0–5 change of direction test.

## Introduction

Soccer is an intermittent team sport characterized by^1^ many high-intensity actions (HIA), such as jumps and sprints, which occur mainly during decisive moments of matches, playing a pivotal role in achieving a tremendous on-field performance^2^. Considering the multidirectional nature of soccer, players must be prepared not only to sprint over linear courses but also to perform rapid change-of-direction (COD)^3–5^, mainly depending on external stimuli (e.g., ball trajectory, opponents’ and teammates’ movements)^6^. In this regard, time-motion analysis has revealed that soccer players perform ~ 100 turns of 90–180° during games^7^, representing ~ 8.5% of the total of HIA^2^. Due to this, optimal players’ preparation to perform such a high neuromuscular demand consistently and effectively is required, so understanding the variables that determine COD performance seems necessary.

The ability to change direction while sprinting is considered an essential component of physical performance in soccer^8^, which allows discriminating players of different playing standards^9^. However, it should be noted that success in these actions is not only based on reaching a higher speed, but the ability to decelerate quickly plays a significant role^10^. This deceleration allows players to decrease step length, apply greater lateral forces to the ground and keep the torso to improve the braking action and consequently, their COD performance^11^. Thus, in kinematic terms, it is not convenient to consider only the peak speed when analyzing the COD ability. Still, other variables, such as peak acceleration and peak deceleration, must be taken into account^12^.

Regarding this, the COD deficit (CODD) has emerged as a suitable approach^13^. Specifically, CODD refers to the additional time that a COD requires when compared to a linear over the same distance^14^ or the difference in velocity between the linear sprint and a COD task of equal distance^15^. Due to the large number of factors that influence COD performance, it seems necessary to know each of them and assess the relationships between them to apply for individualized and specific training programs.

In soccer, the majority of HIA occur unilaterally^16^, not being equal to the implication of both limbs^17^. Due to this, asymmetries are expected to be somewhat expected^18^, which could negatively impact performance^19^. However, there is some controversy on this topic^20^, based on the fact that a given level of asymmetry may be considered functional and necessary^21^. Therefore, inter-leg differences regarding COD ability must be known to optimize soccer players’ preparation. In this sense, Trecroci et al.^22^ observed worse values in COD ability and CODD for the non-preferred leg in youth soccer players. These results were supported by Raya-González et al.^8^ in under-19 soccer players. However, these authors established the preferred leg as the one in which each player obtained the best result.

On the other hand, Rouissi et al.^23^ analyzed the differences in COD ability between preferred and non-preferred legs according to their skill level, observing that the preferred leg performed better in several tests and angles in young soccer players. Despite this, the studies above only considered inter-leg differences in COD ability regarding the time to cover each COD test, and most of them classified the legs in preferred and non-preferred based on their performance in tests (i.e., best vs. worst) but not based on their preference. It is reported that 90% of people exhibit a well-defined right-hand preference whereas 25–45% demonstrate right-leg preference in lower extremity actions^24^. This preference can conduct in asymmetries in performance^24^, and it is vital to further research how the variations can occur. For example, while assessing players in change-of-direction tests, braking with a specific leg may produce a different outcome than braking with another. Thus, it is essential to understand how these leg preferences can affect players differently, which may imply additional information for coaches. Thus, the aim of this study was two-fold: (i) to analyze the variation of peak speed, acceleration, and deceleration measures between using preferred and non-preferred legs while braking during the 5–0–5 COD test; and (ii) to assess the relationships between kinematic measures and CODD.

## Methods

### Study design

A cross-sectional study was conducted to analyze the peak speed, acceleration and deceleration measures obtained by the players while performing the 5–0–5 test using preferred and non-preferred legs while braking. This study was preliminarily approved by the Afyon Kocatepe University ethics committee (Protocol code: 2021/1166, approved on 27.12.2021) and followed the Declaration of Helsinki ethical standards for the study in humans. The participants were informed about the study design, risks, and benefits. After that, they signed an informed consent form.

### Setting and context

The tests were conducted in the middle of the competitive season. Players were tested every day, preceded by a 48-h rest before the assessments. The tests occurred from 3 to 6 p.m., with environmental conditions of 8 °C and relative humidity of 56%. The 5–0–5 test was conducted on an outside synthetic soccer turf pitch. The players answered the Total quality recovery questionnaire (TQR)^25^ before the assessments. Additionally, the players answered the question, “what time did you eat your last meal?”, “how many hours did you sleep this night?” and “what was the day of your last training session or match?”. This information was collected aiming to improve the replicability of the study. It has been found that answers in TQR^25^ are associated with ultimate performance in high-intensity locomotor activities, and sleep can compromise performance^26^. Additionally, they registered their preferred leg, which was used as the independent variable of this study. The answer used for questioning players was, “which is the leg that you prefer for braking?”. Two short runs preceded the questioning with a 180º change of direction in which the players experienced braking with one and the other leg.

### Participants

A priori sample size estimation was performed using the G*Power software (version 3.9.9.6)^27^. For an effect size of 0.5, a power of 0.8, and a *p* value of 0.5, the recommended value was 19. The participants were selected by convenience sampling using a nonprobability strategy. They come from the same soccer team. Twenty male outfield soccer players (21.6 ± 2.0 years old; 8.7 ± 2.3 years of experience; 73.2 ± 6.1 kg; and 174.8 ± 4.5 cm) were voluntarily enrolled in this study. No goalkeepers were part of the study. Eight defenders, six center-midfielders, four wingers, and two forwards were part of the sample. The following eligibility criteria were defined: (i) they were not injured in the last month before the assessments; (ii) they did not report any injury or illness during the day of the assessments or the previous day; (iii) they followed their everyday routines; (iv) no drugs were allowed to take before or during the assessments; and (iv) they rested by 48-h before the assessments (which means that no training session, match, or physical education class was taken during this period).

### Anthropometry

The player’s stature was measured using a stadiometer with a 0.13 cm technical error (SECA Stadiometer 213/Germany), and the body mass was measured using a digital balance (Tanita BC 418 MA analyzer / Tanita Corp., Tokyo, Japan). The stature and body mass was used as the outcome to characterize the participants.

## Protocol of the assessments

The players followed a preliminary warm-up protocol. FIFA 11 + (level 2) was implemented as the standardized protocol for the players. The coach imposed the same warm-up protocol for all the players. The warm-up consisted in running exercises for 8 min (straight ahead, hip out, hip in, circling partner, shoulder contact, quick forward & backward), 10 min of strength, plyometrics, and balance (the alternate bench legs, sideways bench raise & lower hip, hamstrings, single leg stance, squats, lateral jumps) and 2 min of running exercises (across the pitch, bounding, plant & cut). This protocol was proven to improve the COD performance^28,29^. After the FIFA 11 + warm-up, the participants performed three trials of 40-m linear sprint. The players rested for three minutes after the warm-up and sprint trials and before the first 5–0–5 test assessment. The players performed three trials of the 5–0–5 test with the preferred leg, and three trials with the non-preferred leg. They rested for five minutes between a set of three repetitions of 5–0–5 test with one leg and other set of trhee trials with the another leg. And they also rested for 3 min between trials (within the same test).

### The 5–0–5 test

**Figure 1:**
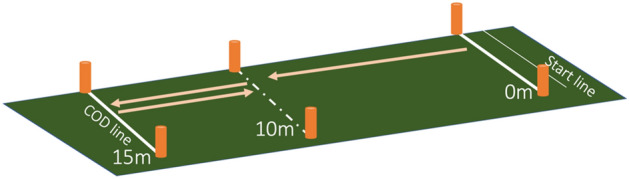
Setup of 5–0–5 test.

The original version of the 5–0–5 test was employed. The test consists of accelerating at the maximum intensity by ten meters, performing a 5-m maximal intensity run followed by a 180° COD (COD line in Fig. 1) and returning for 5-m maximal intensity running (Fig. 1). The players were randomly assigned to two groups. Ten players started the trials by braking with the preferred leg (in the COD line), while ten players started the trials by braking with the non-preferred leg (in the COD line). After completing three attempts, the players changed to the opposite leg.

The players were familiarized with the test since it is part of their regular assessment routines. The players used soccer boats during the runs. The players started at 0.3 m from the first pair of photocells. They started with a staggered stance position, always using the same foot in front. The test used three pairs of photocells (positioned in the starting line, 10-m, and 15-m at COD moment). The single-beamed photocells (Smartspeed; Fusion Sports, Sumner, Australia) were positioned 60 cm from the floor. Players also used the Polar Team Pro (10 Hz, Polar Electro, Kempele, Finland), a Global Positioning System validated and reliable to measure peak speed compared to radar gun^30^. The systems were positioned in a specialized pocked on the player's upper backs. The measures obtained for each trial were: peak speed (km/h), peak acceleration (m/s^2^), and peak deceleration (m/s^2^). The better values (i.e., the highest) obtained for each of the included measures (i.e., peak speed, peak acceleration, and deceleration) for preferred and non-preferred legs were used for further data treatment. Using the information from photocells, it was also possible to calculate the COD deficit (CODD). The CODD was calculated based on the difference of the 10-m COD time subtracted by the 10-m linear sprint time (acceleration phase of the 5–0–5 test). The smallest CODD time (independent of the leg) was obtained for further data analysis.

### Statistical procedures

Descriptive statistics are presented in the form of the average and standard deviation. Data variability within the test (between trials) is presented as the percentage of the coefficient of variation (%CV; for the case of variability reported in the results). The Shapiro–Wilk and Levene’s tests confirmed the normality (*p* > 0.05) and homogeneity (*p* > 0.05) of the sample. Paired t-test was used to compare the best performance in kinematic measures between preferred and non-preferred legs. The standardized effect size of Cohen was used for the comparisons. Additionally, Pearson product-moment correlation was used to test the relationship between the best CODD and peak acceleration, deceleration, and speed variables. The statistical procedures were executed in the SPSS software (version 28.0.0.0, IBM, Chicago, USA) for a *p* < 0.05.

## Results

As contextual information, the players felt 8.0 ± 0.8 in the TQR (“well recovered/somewhat energetic”). Moreover, their latest meal occurred 4.5 ± 0.8 h before the assessments, and on the day of the evaluations, they slept 8.5 ± 0.9 h. They rested 2.4 ± 0.5 days of rest before the assessments.

**Figure 2:**
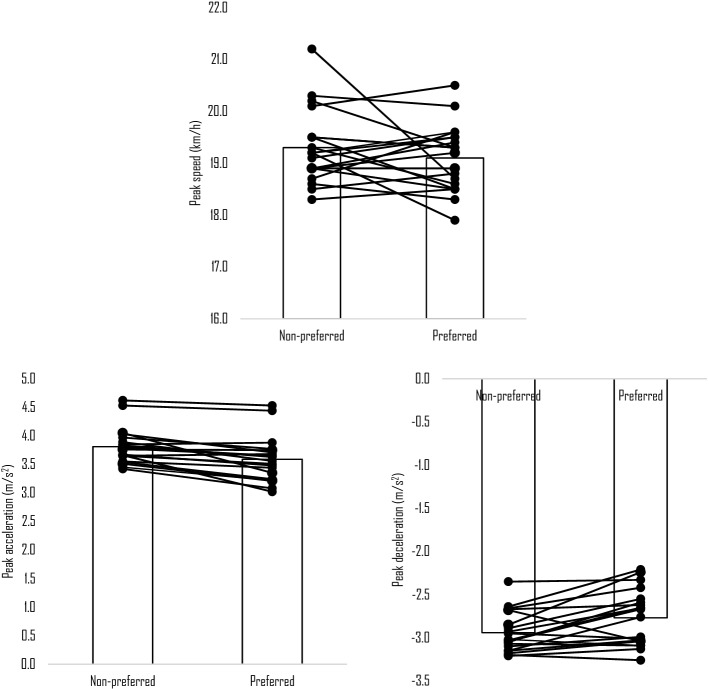
Descriptive statistics and within-players variation of kinematic measures between preferred and non-preferred legs.

Descriptively, the 5–0–5 test time took 2.60 ± 0.12 s for the non-preferred leg and 2.65 ± 0.18 s for the preferred leg. Of the included participants, only two reported the left leg as the preferred one. Figure 2 presents the descriptive statistics of measures for preferred and non-preferred legs. Coefficient of variation for peak acceleration were 3.6 ± 2.4% for preferred and 3.2 ± 1.7% for non-preferred leg, − 4.2 ± 3.7% and − 4.3 ± 3.2% for peak deceleration, 2.3 ± 1.3 and 2.0 ± 1.1% for peak speed. The non-preferred leg presented greater levels of peak speed (19.3 ± 0.7 km/h), peak acceleration (3.8 ± 0.3 m/s^2^) and peak deceleration (− 2.9 ± 0.2 m/s^2^), than preferred leg which presented 19.1 ± 0.6 km/h, 3.6 ± 0.4 m/s^2^, and − 2.8 ± 0.3 m/s^2^, respectively. Comparisons revealed a significantly greater peak acceleration (+ 0.22 m/s^2^; t = 5.131; *p* < 0.001; d = 0.581) and deceleration (+ 0.17 m/s^2^; t = − 3.266; *p* = 0.004; d = − 0.586) for the non-preferred leg. While there were no significant differences found between legs for peak speed (t = 1.142; *p* = 0.268; d = 0.298).

**Figure 3:**
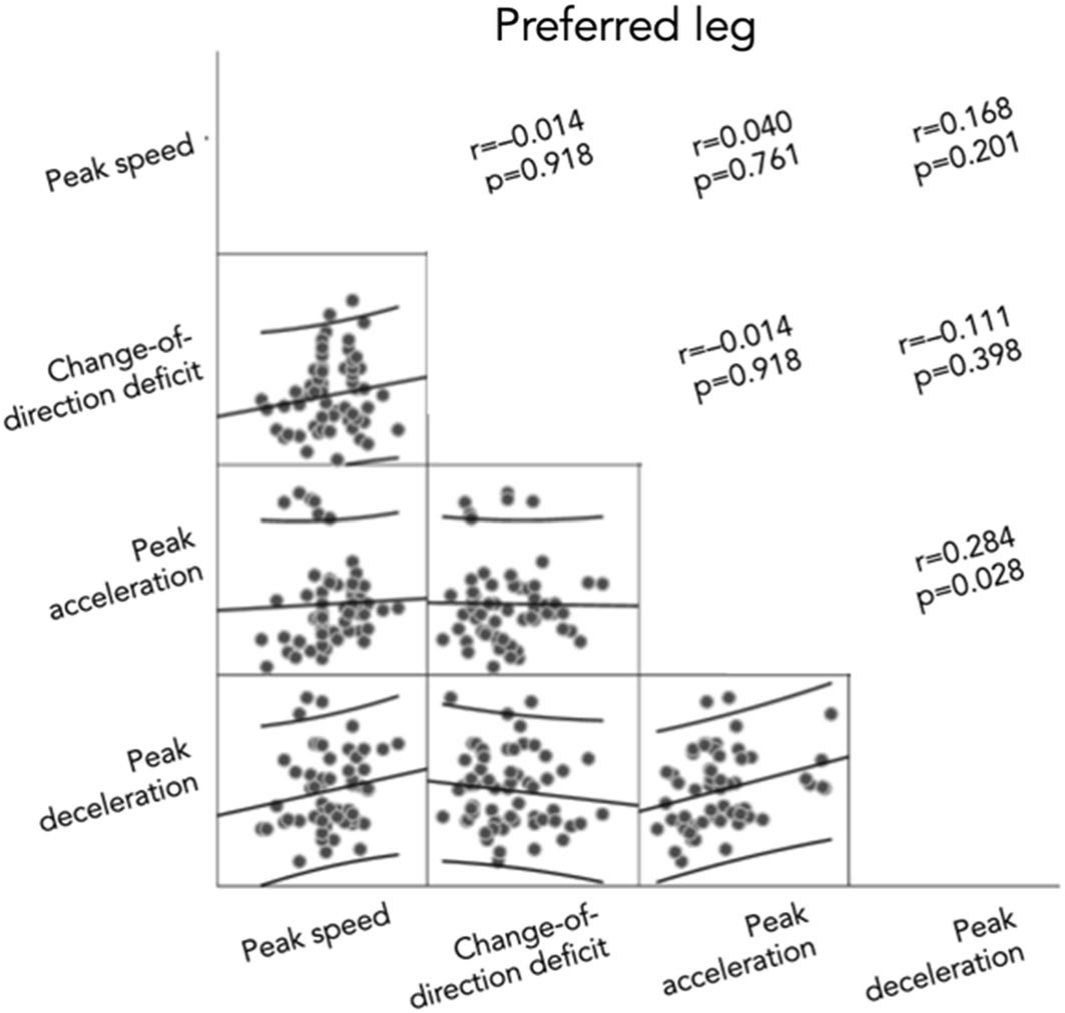
Correlation matrix between measures for preferred leg.

**Figure 4:**
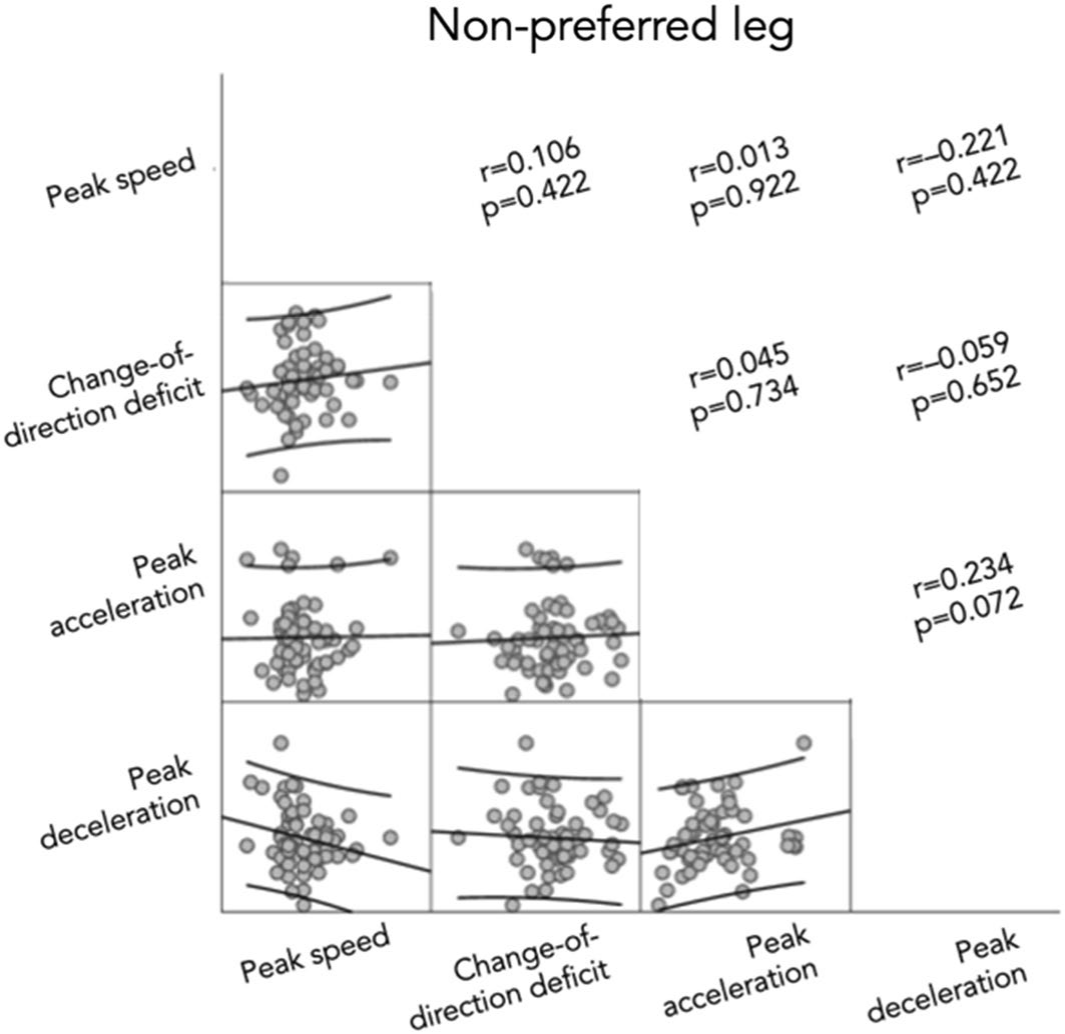
Correlation matrix between measures for non-preferred leg.

Relationships between measures were tested and can be found in Figs. 3 and 4. No significant correlations were found between CODD and peak accelerations (r = − 0.014, [− 0.193; 0.166]), peak decelerations (r = − 0.052, [− 0.229; 0.128]) or peak speed (r = 0.118, [− 0.063; 0.291]). There were also no significant correlations for the non-preferred leg, between CODD and peak accelerations (r = 0.045, [− 0.212; 0.295]), peak decelerations (r = − 0.059, [− 0.308; 0.198]), or peak speed (r = 0.106, [− 0.153; 0.349]), or for the preferred leg (while braking) between CODD and peak accelerations (r = − 0.014, [− 0.266; 0.241]), peak decelerations (r = − 0.111, [− 0.354; 0.148]), and peak speed [r = 0.150, [− 0.109; 0.388]).

## Discussion

The aim of this study was two-fold: (i) analyze the variation of kinematic measures between using preferred and non-preferred legs while braking during the 5–0–5 change of direction test; and (ii) test the relationships between kinematic measures, and change-of-direction deficit (CODD). The main results showed a significantly greater peak acceleration and deceleration for the non-preferred leg than for the preferred leg.

Considering the first aim, and independently of the results, some studies showed a tendency for athletes to perform a better change of direction movements with a specific limb^31–35^. In the particular case of soccer, this results from an asymmetry associated with the repeated kicking movements with a preferred limb^36,37^ which contributed to the directional dominance^31,34^ and consequently for the development of one side only (dominant)^36^.

For instance, Dos’Santos et al.^38^ observed higher asymmetries in youth female netball athletes for CODD compared to 5–0–5 completion times. Thus, it seems clear that team sports, such as soccer, would present a better performance in a change of direction movements in a particular direction, suggesting that coaches and their staff should assess asymmetries in this type of movement to provide better training prescription designs^38^.

A previous study attributed the differences in the 5–0–5 change of direction test to the different braking strategies to change direction^39^. Some justification could be associated with higher horizontal braking forces in the penultimate foot contact, pointed out by previous studies as a significant factor for better 180° turns^40,41^. Another study associated better performance due to higher storage and utilization of elastic energy as the muscle lengthens under an eccentric load during the braking phase before the change of direction^42^. Indeed, it had been suggested that higher eccentric strength allows the change of direction with a shorter braking time and, consequently a faster transition to the propulsive phase of the next acceleration^42^.

Although the present study did not address strength, it had been shown that better performance in changing direction movements was associated with higher isometric strength. This strength is fundamental to keeping a lower body position during braking, turning, and acceleration^43^. This may explain the results of the present study because the non-preferred leg is the one that provides support for the kicking movements, and thus it is expected more isometric strength.

Moreover, another study^38^ justified the differences with hypothetical joint coordination, which was not addressed in the present study. Even so, the present study's findings seem to be in opposition once the better performance was found for the non-preferred leg. Thus, it seems essential to study braking strategies and legs/limbs asymmetries to develop knowledge on this topic.

Regarding the second aim of this study, where no significant correlations between CODD and peak accelerations, peak decelerations, or peak speed were found, this seems to be the first study to analyze such associations.

In this sense, previous research found associations between the 5–0–5 change of direction test and CODD^14,38,44–46^, which means that athletes with small CODD tended to perform better in the 5–0–5 change of direction. Furthermore, some studies^14,44–46^ found non-significant associations between CODD and 10-m sprint times for both preferred and non-preferred legs. In contrast, a large and positive association was found between 5–0–5 and 10-m sprint times for both preferred and non-preferred legs. Surprisingly, the results of the present study did not seem to align with the previous studies. Still, it is important to notice that different metrics were used since the current study used peak accelerations, decelerations, and speed. Thus, more studies are needed to confirm such results through acceleration-based metrics. Another suggestion is to use the different peaks for acceleration/deceleration during the different phases of the 5–0–5 change of direction test.

The present study contains some limitations that should be acknowledged. First, the study's cross-sectional nature does not allow an understanding of causal relationships between the measures. Second, the small sample size and the context of the data collected do not allow a proper generalization of the results. Third, as suggested in previous research^44,47^, the unilateral strength, agility, and balance tests seem to be required for a complete analysis of the CODD, acceleration, deceleration, and peak speed movements. Finally, the motor knowledge level can affect performance since motor ability cannot be good enough to ensure the best performance in a change of direction. Future studies should consider identifying the motor knowledge of the players, increasing the sample size while diversifying the teams observed, and analyzing qualitative measures related to the movement while changing direction. Despite the previous limitations, the present study showed that the non-preferred leg performed better in changing direction moments. Such information can be considered for coaches and strength and conditioning staff members to develop training sessions to develop such characteristics in the preferred leg.

## Conclusions

The main results showed a significantly greater peak acceleration and deceleration for the non-preferred leg than for the preferred leg. This study indicates as a practical implication that the preferred and non-preferred leg must be analyzed differently since they are different in the peak speed and peak acceleration and deceleration. Thus, while implementing the test, coaches must require the players to change direction with different feet in front. Another finding from our research is that CODD seems independent of leg preference and the kinematic measures of a 5–0–5 change of direction test.

## Data Availability

The datasets generated during and analyzed during the current study are available from the corresponding author upon reasonable request.
